# The DNA sensor AIM2 mediates psoriasiform inflammation by inducing type 3 immunity

**DOI:** 10.1172/jci.insight.171894

**Published:** 2024-10-01

**Authors:** Timna Varela Martins, Bruno Marcel Silva de Melo, Juliana Escher Toller-Kawahisa, Gabriel Victor Lucena da Silva, Conceição Elidianne Aníbal Silva, Isadora Marques Paiva, Gabriel Azevedo Públio, Marcos Henrique Rosa, Cacilda da Silva Souza, Dario Simões Zamboni, Fernando Q. Cunha, Thiago Mattar Cunha, Bernhard Ryffel, Nicolas Riteau, José C. Alves-Filho

**Affiliations:** 1 Department of Pharmacology, Ribeirão Preto Medical School, and; 2 Center for Research in Inflammatory Diseases, Ribeirão Preto Medical School, University of Sao Paulo, Ribeirão Preto, Sao Paulo, Brazil.; 3 Immune Health Laboratory, Regulation of host responses and immune health, IRL2029, French National Centre for Scientific Research (CNRS) and Ribeirão Preto Medical School (FMRP) of the Sao Paulo University (USP), Sao Paulo, Brazil.; 4 INEM, CNRS, UMR7355 and University, Orleans, France.; 5 Dermatology division, Ribeirão Preto Clinical Hospital, Sao Paulo, Brazil.; 6 Department of Cell Biology, Ribeirão Preto Medical School, University of Sao Paulo, Ribeirao Preto, Sao Paulo, Brazil.

**Keywords:** Inflammation, Skin

## Abstract

Psoriasis is a chronic and recurrent inflammatory skin disease characterized by abnormal proliferation and differentiation of keratinocytes and activation of immune cells. However, the molecular driver that triggers this immune response in psoriatic skin remains unclear. The inflammation-related gene absent in melanoma 2 (AIM2) was identified as a susceptibility gene/locus associated with psoriasis. In this study, we investigated the role of AIM2 in the pathophysiology of psoriasis. We found elevated levels of mitochondrial DNA in patients with psoriasis, along with high expression of AIM2 in both the human psoriatic epidermis and a mouse model of psoriasis induced by topical imiquimod (IMQ) application. Genetic ablation of AIM2 reduced the development of IMQ-induced psoriasis by decreasing the production of type 3 cytokines (such as IL-17A and IL-23) and infiltration of immune cells into the inflammatory site. Furthermore, we demonstrate that IL-17A induced AIM2 expression in keratinocytes. Finally, the genetic absence of inflammasome components downstream AIM2, ASC, and caspase-1 alleviated IMQ-induced skin inflammation. Collectively, our data show that AIM2 is involved in developing psoriasis through its canonical activation.

## Abstract 2

We demonstrated that AIM2 functions through its canonical activation pathway involving ASC and caspase-1 inducing the generation of the IL-23/IL-17 immune axis required for developing psoriasis.

## Introduction

Psoriasis is a chronic and recurrent inflammatory skin disease that affects approximately 2% of the worldwide population (1). Its multifactorial etiology results from a complex interaction between genetic and environmental factors. Psoriatic plaques are characterized by abnormal proliferation and differentiation of keratinocytes (KCs), leading to epidermal hyperplasia and a disordered activation of immune cells, resulting in massive inflammatory cell infiltration in the dermis and epidermis (2, 3). The development and maintenance of psoriasis depend on various cells, including the immune cell system, such as DCs, T lymphocytes, and neutrophils as well as nonimmune cells, such as KCs (4). However, the trigger for this immune response remains unclear.

Absent in melanoma 2 (AIM2) is an intracellular double-stranded DNA (dsDNA) receptor that senses dsDNA in the cytosol and is a critical host defense mechanism against bacterial and viral infections (5). The binding of dsDNA to AIM2 triggers the assembly of an inflammasome complex, resulting in ASC (encoded by the gene *PYCARD*) oligomerization and the subsequent recruitment and activation of caspase-1 (encoded by the gene *CASP1*); this cleaves pro–IL-1β and pro–IL-18 cytokines into their mature forms IL-1β and IL-18, resulting in a strong inflammatory response (6–8). AIM2 was identified as a susceptibility gene/locus associated with psoriasis (9). Moreover, AIM2 expression is increased in KCs from skin lesions of patients with psoriasis, and its activation in cultured KCs leads to IL-1β release, suggesting that AIM2 is involved in the pathogenesis of psoriasis (10). However, the contribution of AIM2 in modulating the immune response during the development and persistence of psoriasis has not yet been elucidated.

In this study, we conducted a systematic and integrative analysis of different public transcriptomic datasets of human psoriatic skin. Our analysis showed that pathways related to cytosolic sensors of pathogen-associated DNA were among the main differentially expressed genes. Accordingly, we found a significant increase in AIM2 expression within the epidermis of lesioned skin in human psoriasis and in psoriasiform skin inflammation induced by the topical application of imiquimod (IMQ) in mice. Interestingly, deficiency of AIM2 reduced the development of IMQ-induced psoriasis by decreasing the production of type 3 cytokines (such as IL-17A and IL-23) and infiltrating immune cells into the inflammatory site. Therefore, our study demonstrates the critical role of the AIM2-dependent inflammasome in the pathogenesis of skin psoriatic inflammation and proposes AIM2 as a promising therapeutic target for psoriasis.

## Results

### AIM2 is highly expressed in psoriatic lesions and is required to induce psoriasiform inflammation.

**Figure 1:**
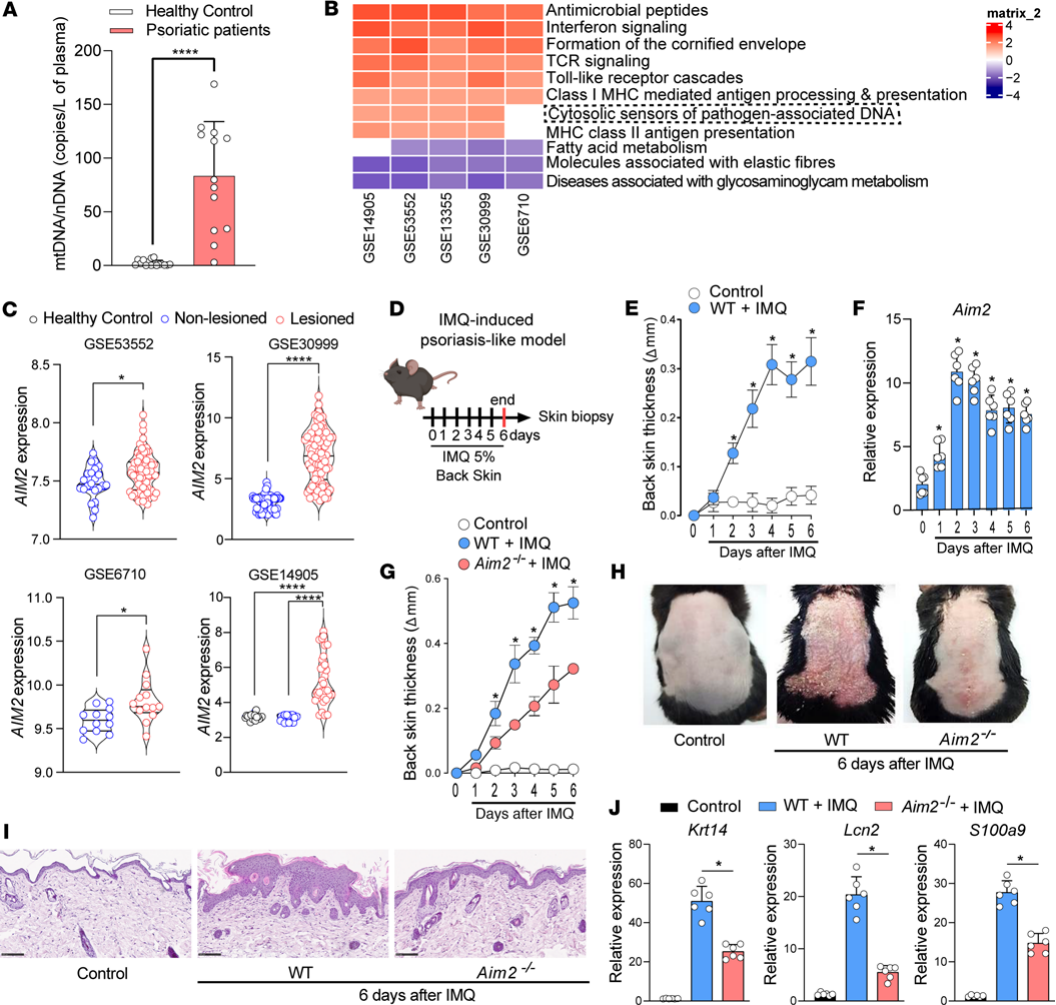
AIM2 is required for the development of experimental psoriasis. (**A**) Plasma concentration of mtDNA (mtDNA/nDNA copies/μL) in healthy individuals (*n* = 16) and patients with psoriasis (*n* = 13). (**B**) Heatmap showing the differentially expressed genes (DEGs) between lesioned and nonlesioned psoriatic skin from 5 databases, using a blue-to-red continuous color scale. (**C**) AIM2 expression in skin biopsy samples from healthy controls, nonlesioned, and lesioned sites from patients with psoriasis, as determined by transcriptome analysis in 4 published datasets. (**D**) Schematic illustration of psoriasis-like model induction using IMQ, applied for 6 days on the backs of the mice. (**E**) Daily measurement of back-skin thickness after topical IMQ application in WT mice. Untreated mice served as controls (*n* = 6 per group). (**F**) Time course of relative *Aim2* mRNA expression in IMQ-treated and untreated WT mice, normalized to *Gapdh* in whole-skin lysates, evaluated by qPCR (*n* = 6 per group). (**G**) Back skin thickness (Δ) measured by caliper daily after IMQ application in WT and *Aim2^–/–^* mice. Untreated WT mice were used as controls (*n* = 6 per group). (**H**) Representative pictures of inflammation in shaved back skin. (**I**) H&E staining of back skin sections before IMQ. Naive WT mice were used as controls. Images were acquired at 20× magnification. Scale bar: 100 μm (*n* = 6 per group). (**J**) Relative mRNA expression of *Kr14*, *Lcn2*, and *S100a9* genes in whole skin lysates evaluated by qPCR and normalized to *Gapdh* (*n* = 6 per group). Data are representative of 2–3 independent experiments and are shown as mean ± SEM. Statistical significance was evaluated by 2-tailed unpaired Student’s *t* test in **A**, 1-way ANOVA with Bonferroni post hoc test in **C** and **J**, and 2-way ANOVA followed by Bonferroni’s post hoc test in **E**–**G**. *P < 0.05, ***P < 0.001, ****P < 0.0001.

Damaged mitochondria released mitochondrial DNA (mtDNA) can activate AIM2 inflammasomes (11–13). To explore the role of AIM2 in the clinical cutaneous manifestations of psoriasis, we began by analyzing circulating cell-free mtDNA levels in patients with psoriasis. Interestingly, we found that mtDNA levels are elevated in cell-free plasma from patients with psoriasis (Figure 1A). We then analyzed publicly available gene array data from 5 different NCBI Genome Expression Omnibus (GEO) databases (GSE14905, GSE53552, GSE13355, GSE30999, GSE6710; https://www.ncbi.nlm.nih.gov/geo/) and investigated the global gene signature between lesioned and nonlesioned skin from patients with psoriasis (14–18). We found that the cytosolic sensors of pathogen-associated DNA were one of the top upregulated pathways in these databases (Figure 1B). We then evaluated *AIM2* expression in skin biopsies from healthy volunteers and compared it with lesioned and nonlesioned areas of patients with psoriasis in 4 different NCBI GEO databases (14, 15, 17, 18). Strikingly, *AIM2* mRNA levels in injured psoriasis skin increased as compared with normal and uninjured psoriasis skin (Figure 1C). To further explore the importance of AIM2 in psoriasis, we used the psoriasiform skin inflammation model triggered by the topical application of IMQ, a TLR7/8 agonist widely used to induce a murine model of psoriasis (19). As expected, IMQ-treated WT mice showed a significant increase in back skin thickness compared with the untreated group (Figure 1, D and E). Consistent with data from patients with psoriasis, we found that *Aim2* mRNA increased significantly in the skin of mice, particularly on days 2 and 3, following topical IMQ application (Figure 1F). To investigate the involvement of AIM2 in psoriasis pathophysiology, we used AIM2-deficient mice (*Aim2*^–/–^). Interestingly, *Aim2*^–/–^ mice showed a marked reduction in back skin thickness and macroscopic inflammation compared with IMQ-treated WT mice (Figure 1, G and H). Histological analysis of back skin cross-sections from *Aim2*^–/–^ mice after IMQ application revealed reduced epidermal hyperplasia and a reduction in inflammatory cells in the dermis compared with IMQ-treated WT mice (Figure 1I). This was further supported by decreased gene expression of KC activation markers *Krt14* (keratin 14)*, Lcn2* (lipocalin 2)*,* and *S100a9* (S100 calcium binding A9) (Figure 1J). Of note, no evident differences in the development or homeostasis of the epidermis and dermis were observed between *Aim2*^–/–^ and WT mice (Supplemental Figure 1; supplemental material available online with this article; https://doi.org/10.1172/jci.insight.171894DS1). Collectively, these findings demonstrate that AIM2 is highly expressed in the skin of patients with psoriasis and mice following IMQ treatment. Furthermore, AIM2 signaling exacerbates the pathophysiology of IMQ-induced psoriasis–like skin inflammation.

### Deficiency of AIM2 reduces experimental psoriasis-like skin inflammation.

**Figure 2:**
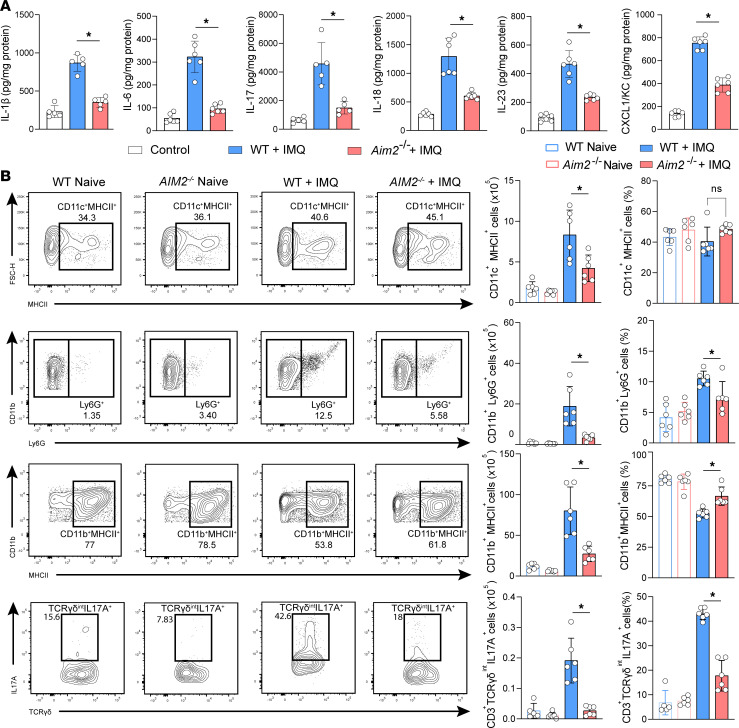
AIM2 promotes psoriasiform skin inflammation. (**A**) IL-1β, IL-6, IL-17A, IL-18, IL-23, and CXCL1/KC concentration in skin homogenates of WT and *Aim2^–/–^* mice determined by ELISA 6 days after IMQ treatment. Untreated WT mice were used as controls (*n* = 6 per group). (**B**) Representative flow cytometry dot plots, absolute numbers, and frequency of CD11c^+^MHCII^+^ DCs, CD11b^+^Ly6G^+^ neutrophils, CD11b^+^Ly6G^–^Ly6C^–^MHCII^+^ macrophages, and IL-17–producing γδ T cells (CD3^+^TCRγδ^int^) gated on live CD45^+^ cells extracted from ear skin (*n* = 5–6 per group). Data are representative of 2–3 independent experiments and are shown as mean ± SEM. Statistical significance was evaluated by 1-way ANOVA with Bonferroni post hoc test. *P < 0.05.

Next, we sought to investigate the pathophysiological relevance of AIM2 expression to establish psoriasis-like skin inflammation. To this end, we evaluated the expression of cytokines and chemokines associated with psoriasis. We found that the absence of AIM2 significantly reduced the protein expression of IL-1β, IL-6, IL-17A, IL-18, IL-23, and CXCL1/KC compared with IMQ-treated WT mice (Figure 2A). These inflammatory mediators are involved in the recruitment or activation of immune cells, leading to inflamed skin (20). We found that AIM2 deficiency leads to a reduction in the absolute numbers of DCs (CD11c^+^MHCII^+^), neutrophils (CD11b^+^Ly6G^+^), macrophages (CD11b^+^Ly6G^–^Ly6C^–^MHCII^+^), and IL-17–producing γδ T cells (CD3^+^TCRγδ^int^IL-17A^+^) in the IMQ-treated skin (Figure 2B and Supplemental Figure 2). These data suggest that AIM2 is involved in the modulation of inflammatory mediators affecting the infiltration of immune cells into the inflamed skin.

### AIM2 is overexpressed in the epidermis of psoriatic skin and is regulated by IL-17A.

**Figure 3:**
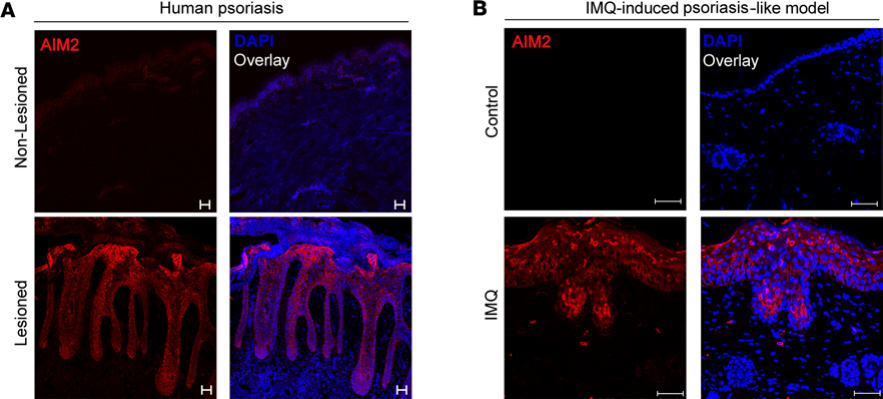
Increased AIM2 expression in keratinocytes in psoriasis. (**A**) Immunofluorescence imaging of frozen sections from skin biopsies of patients with psoriasis covering nonlesioned and lesioned sites (acquired at 20× magnification) (*n* = 3 per group). (**B**) Back skin sections from WT mice after 6 days of IMQ treatment (acquired at 40× magnification) stained for AIM2 (red) and DAPI (blue). Scale bars: 50 μm.

**Figure 4:**
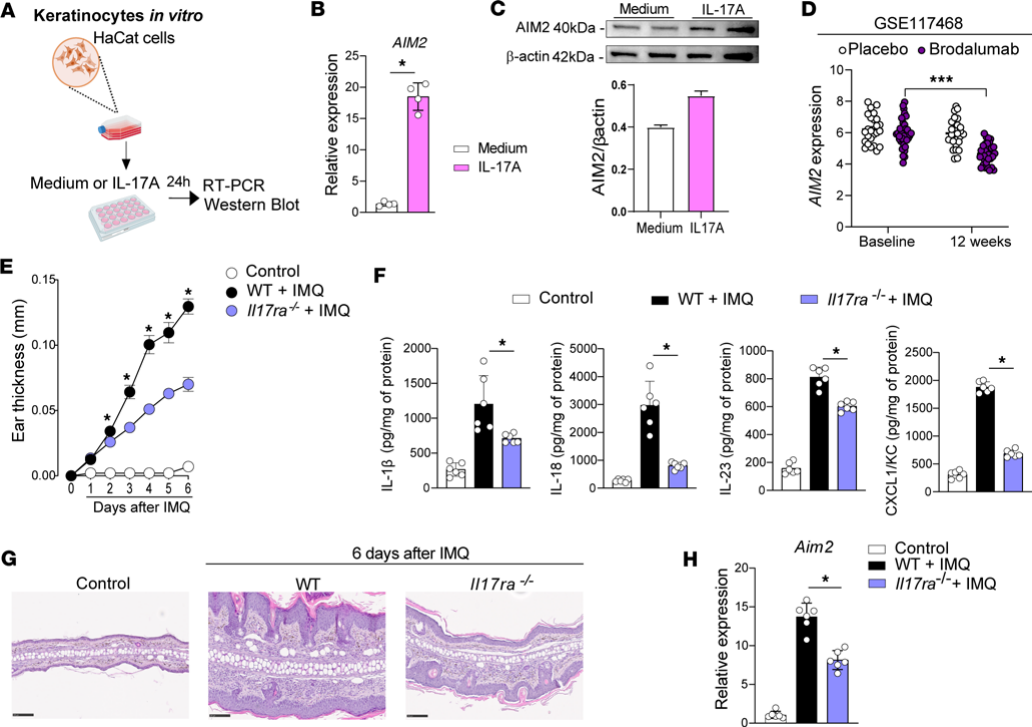
IL-17A regulates AIM2 expression in psoriasis-like disease models. (**A**) Schematic illustration of HaCaT cells activated with IL-17A for 24 hours. (**B** and **C**) Relative AIM2 mRNA expression (*n* = 4 per group) (**B**) and AIM2 protein levels (*n* = 2 per group) (**C**) determined by qPCR or Western blot, respectively. (**D**) *AIM2* expression in the skin before and after brodalumab treatment in human psoriasis from GSE117648 database. (**E**–**H**) IMQ was applied for 6 consecutive days on the backs of WT and *Il17ra*^–/–^ mice (*n* = 6 per group). (**E**) Ear thickness was measured daily. (**F**) IL-1β, IL-18, IL-23, and CXCL1/KC concentrations were measured in the back skin by ELISA. (**G**) H&E staining of back skin sections in WT and *Il17ra*^–/–^ mice treated with IMQ (acquired at 20× magnification). Untreated WT mice were used as controls. Scale bars: 100 μm. (**H**) *Aim2* mRNA expressions in whole-skin lysates of WT and *Il17ra*^–/–^ mice after IMQ treatment evaluated by qPCR and normalized to *Gapdh*. Untreated WT mice were used as controls. Data are representative of 2–3 independent experiments and are shown as mean ± SEM. Statistical significance was evaluated by 2-tailed unpaired Student’s *t* test in **B**, 2-way ANOVA followed by Bonferroni’s post hoc test in **D** and **E**, and 1-way ANOVA with Bonferroni post hoc test in **F** and **H**. *P < 0.05 and ***P < 0.001.

We next evaluated AIM2 expression using immunofluorescence by confocal microscopy in the skin of patients with psoriasis and IMQ-treated mouse skin. Our data indicate that AIM2 is predominantly expressed in the epidermis of the lesioned skin of patients with psoriasis or mice undergoing the IMQ-induced psoriasis model (Figure 3, A and B). Since IL-17A is a crucial mediator in the pathogenesis of psoriasis, inducing the proliferation and secretion of inflammatory mediators by KCs (21, 22), we next investigated whether IL-17A could modulate AIM2 expression in KCs. To this end, we cultured a human KC cell line (HaCaT) in the presence or absence of recombinant IL-17A (Figure 4A). Activation of HaCaT cells with IL-17A resulted in a significant upregulation of *AIM2* gene expression (Figure 4B). Consistently, immunoblot analysis confirmed the increased protein expression of AIM2 after activation of HaCaT cells with IL-17A (Figure 4C). We also observed that IFN-γ can induce the expression of *AIM2* in HaCaT cells but is less effective than IL-17A (Supplemental Figure 3). We next analyzed a public GEO database (GSE117468) (23) to assess the relative expression of *AIM2* mRNA in lesioned skin biopsies from patients with psoriasis who were treated with brodalumab, a human monoclonal antibody targeting the receptor subunit IL-17RA (24). Skin biopsies of the lesions were obtained from patients before treatment (baseline) and 12 weeks after the s.c. injection of brodalumab or placebo. Details of the clinical trial design and results have been previously reported (23). Notably, we found a significant reduction of *AIM2* mRNA in skin biopsies from patients after treatment with brodalumab (Figure 4D). We then took advantage of the IMQ-induced psoriasis–like model and determined whether the loss of IL-17A signaling in IL-17RA–deficient mice (*IL17ra^–/–^*) would affect AIM2 expression in the skin. As already described in the literature, *Il17ra^–/–^* mice exhibited markedly reduced skin inflammation, thickness, and epidermal hyperplasia as well as lower levels of IL-1β, IL-18, IL-23, and CXCL1/KC after the daily topical application of IMQ compared with the WT mice (Figure 4, E–G). Interestingly, IMQ-treated *IL17ra^–/–^* mice showed lower gene expression of AIM2 in the skin than WT mice (Figure 4H). Altogether, our findings show that AIM2 is overexpressed in the epidermis of psoriatic skin and IL-17A regulates its expression in KCs.

### AIM2 activation directly induces psoriasiform skin inflammation.

**Figure 5:**
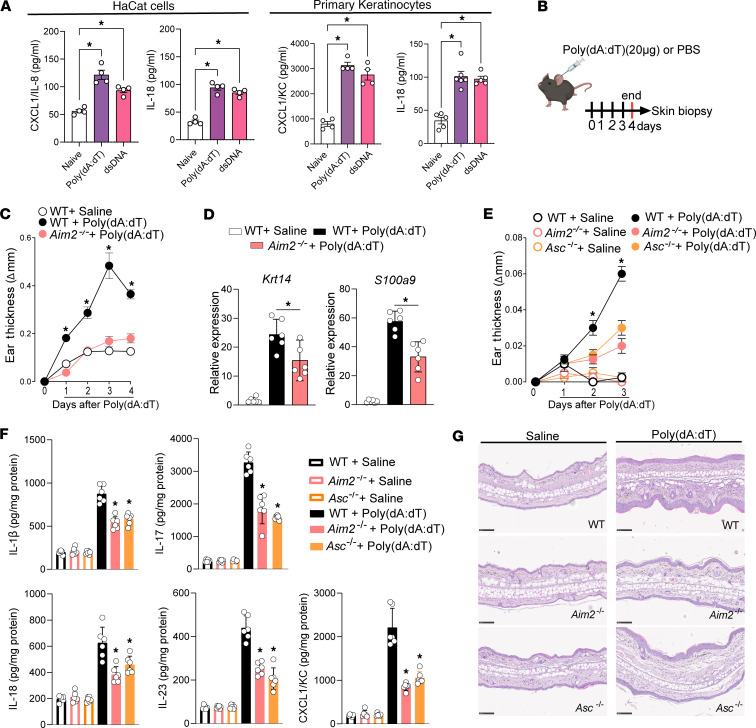
Intradermal injection of AIM2 ligand induces psoriasis-like inflammation. (**A**) HaCaT cells and primary keratinocytes were activated with AIM2 agonist poly(dA:dT) (500 ng/mL) or dsDNA (500 ng/mL) for 24 hours, and CXCL8/IL-8 and IL-18 were measured in the cell supernatant, respectively (*n* = 4–5 per group). (**B**–**G**) Poly(dA:dT) (20 μg) or saline was injected intradermally in the ears of WT, *Aim2^–/–^*, and *Pycard*^–/–^ mice for 3 or 4 consecutive days as indicated. (**B**) Schematic of the experimental model. (**C**) Time course of changes in the ear skin thickness (Δ) measured by caliper (*n* = 4–6 per group). (**D**) Relative mRNA expressions of *Krt14* and *S100a9* normalized to *Gapdh* in whole skin lysates evaluated by qPCR (*n* = 6 per group). (**E**) Time course of changes in the ear skin thickness (Δ) measured by caliper (*n* = 4 per group). (**F**) IL-1β, IL-17, IL-18, IL-23, and CXCL1/KC levels in skin homogenates determined by ELISA (*n* = 6 per group). (**G**) Representative H&E staining of mouse ear skin sections (acquired at 20× magnification). Scale bar: 100 μm (*n* = 6 per group). Data are representative of 2 independent experiments and are shown as mean ± SEM. Statistical significance was evaluated by 1-way ANOVA followed by Bonferroni’s post hoc test in **A**, **D**, and **F**, and 2-way ANOVA with Bonferroni post hoc test in **C** and **E**. *P < 0.05.

We then investigated whether a direct activation of AIM2 could trigger psoriasiform skin inflammation. We assessed the ability of poly(dA:dT), an AIM2 ligand, or dsDNA extracted from KCs to induce the release of inflammatory mediators by KCs. Our results demonstrate that both poly(dA:dT) and dsDNA could induce the production of CXCL1/CXCL8 and IL-18 by HaCaT cells and primary murine KCs (Figure 5A). Subsequently, we performed intradermal injections of poly(dA:dT) or saline for 4 consecutive days to evaluate skin inflammation (Figure 5B). The injection of poly(dA:dT) led to a significant increase in skin thickness in WT mice compared with saline-treated mice. Notably, *Aim2*^–/–^ mice showed a significant reduction in ear thickness after poly(dA:dT) injection (Figure 5C), accompanied by a decrease in *Krt14* and *S100a9* gene expression (Figure 5D). To investigate whether the proinflammatory effect observed upon direct AIM2 activation depends on the inflammasome component, we administered poly(dA:dT) to ASC-deficient mice (*Pycard*^–/–^). We found a significant reduction in ear thickness in poly(dA:dT)-treated *Pycard*^–/–^ mice, similar to that seen in *Aim2*^–/–^ mice (Figure 5E). Furthermore, there was a decrease in the production of inflammatory mediators, IL-1β, IL-17A, IL-18, IL-23, and CXCL1/KC, in poly(dA:dT)-treated *Aim2*^–/–^ and *Pycard*^–/–^ mice (Figure 5F). Consistently, histological cross-sections of the ear showed decreased epidermal hyperplasia with a lower accumulation of inflammatory cells in the dermis of poly(dA:dT)-treated *Aim2*^–/–^ and *Pycard*^–/–^ mice as compared with poly(dA:dT)-treated WT mice (Figure 5G). Together, these findings suggest the role of the AIM2 inflammasome pathway in triggering skin inflammatory responses observed during psoriasis.

### AIM2 inflammasome is necessary for the induction of psoriasis-like skin inflammation.

**Figure 6:**
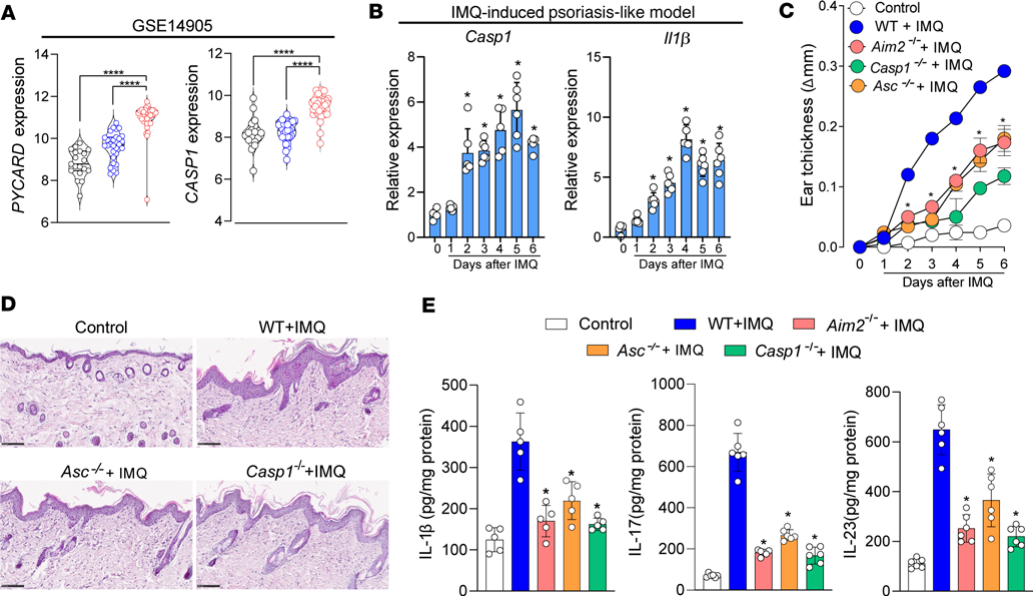
ASC and caspase-1 are required for the development of IMQ-induced psoriasis in mice. (**A**) *PYCARD* and *CASP1* gene expression in skin biopsy samples from healthy control and nonlesioned or lesioned sites from patients with psoriasis evaluated by analysis of a published transcriptomic dataset (GSE14905). (**B**–**E**) WT, *Aim2^–/–^*, *Pycard*^–/–^, and *Casp1^–/–^* mice after 6 days of IMQ treatment. Untreated WT mice were used as controls. (**B**) Kinetics of relative mRNA expression of *Casp1* and *Il1b* in the whole skin lysates from IMQ-treated WT mice normalized to *Gapdh* evaluated by qPCR (*n* = 6 per group). (**C**) Time course of changes in the back skin thickness (Δ) measured daily after topical IMQ application by caliper (*n* = 6 per group). (**D**) Representative H&E staining from back skin sections. Images were acquired at 20× magnification. Scale bar: 100 μm (*n* = 6 per group). (**E**) IL-1β, IL-17, and IL-23 concentration in skin homogenates determined by ELISA (*n* = 6 per group). Data are representative of 2 independent experiments and are shown as mean ± SEM. Statistical significance was evaluated by 1-way ANOVA with Bonferroni post hoc test in **A** and **E**, and 2-way ANOVA followed by Bonferroni’s post hoc test in **B** and **C**. *P < 0.05 and ****P < 0.0001.

We finally investigated whether the AIM2 function depends on inflammasome components during psoriasis. First, we observed a significant increase in the expression of *PYCARD* (encoding for ASC) and *CASP1* (encoding for caspase-1) in the injured skin of patients with psoriasis compared with nonlesioned or healthy control skin groups (Figure 6A). A strong upregulation of *Casp1* and *Il1b* expression was also found in the IMQ-induced psoriasis mouse model (Figure 6B). To investigate the contribution of ASC and caspase-1, we used mouse strains deficient for ASC (*Pycard^–/–^*) or caspase-1 (*Casp1^–/–^*). The absence of ASC or caspase-1 led to reduced skin hyperplasia as measured by dorsal skin thickness, similar to what is observed in the absence of AIM2 (Figure 6C). These data were supported by H&E staining of the dorsal skin sections, which showed reduced epidermal thickening and acanthosis in IMQ-treated *Pycard*^–/–^ and *Casp1^–/–^* mice as compared with IMQ-treated WT mice (Figure 6D). Importantly, we show that ASC or caspase-1 deficiency prevented the production of proinflammatory mediators such as IL-1β, IL-17, and IL-23 (Figure 6E). Consistently, the absence of IL-1R attenuated skin thickening and production of IL-1β, IL-17, and IL-23 in IMQ-induced psoriasis model mice (Supplemental Figure 4). Altogether, these data demonstrate that the AIM2 inflammasome pathway promotes the pathophysiology of IMQ-induced skin inflammation in an inflammasome-dependent manner.

## Discussion

AIM2 activation triggers the formation of the inflammasome, an innate immune complex that drives the activation of inflammatory pathways, resulting in the secretion of IL-1β and IL-18 (6–8). Both experimental and clinical evidence highlight the crucial role of these proinflammatory cytokines in the pathogenesis and progression of psoriatic skin inflammation (25, 26). Previous studies have highlighted AIM2 activation in the pathophysiology of several autoimmune and inflammatory diseases, such as systemic lupus erythematous, colitis, and type 2 diabetes (13, 27, 28). Moreover, the whole-exosome SNP array identified the *AIM2* gene as a new susceptibility locus for psoriasis (9). However, it was still unclear whether AIM2 participates in developing psoriasis. Here, we found a high expression of AIM2 in both the human psoriatic epidermis and a mouse model of psoriasis induced by topical IMQ application. Moreover, we provide evidence that genetic AIM2 deficiency prevents IMQ-induced skin inflammation through reduced production of proinflammatory cytokines and chemokines, which decrease the migration and activation of inflammatory cells in the skin.

KCs constitute the majority of cells in the skin epidermis, and during psoriasis, these cells have abnormal proliferation and differentiation, leading to epidermal hyperplasia (29). Using the IMQ-induced psoriasis model, we found that *Aim2* deficiency reduces mRNA expression of the KC markers *Lcn2*, *Krt14*, and *S100a9*. Several studies have revealed that IL-17A is an essential cytokine for activating KCs, leading to a positive feedback loop that induces the production of additional cytokines and chemokines, amplifying inflammation, and one that IL-17A plays a central role in the pathogenesis of psoriasis (20, 21, 24). Our study demonstrates that IL-17A directly upregulated AIM2 expression in KCs in vitro. In addition, patients treated with the IL-17A receptor inhibitor brodamulab show decreased AIM2 expression in the skin, supporting that IL-17 mediates AIM2 upregulation. Finally, IMQ-treated *Il17ra*^–/–^ mice display lower *Aim2* expression than IMQ-treated WT mice. Together, these findings suggest that AIM2 is required for the activation/proliferation of KCs and that its expression is modulated by IL-17A, which leads to positive feedback shaping the psoriasiform skin inflammation.

AIM2 activation occurs mainly after the emergence of self- or foreign DNA in the cytosol, where it is perceived as a danger signal to alert the host by promoting inflammation (30). Considering this, we investigated the levels of circulating DNA and found that mtDNA levels are elevated in plasma from patients with psoriasis but decreased upon treatment with brodamulab. Cao et al. (31) proposed that neutrophil extracellular traps (NETs) from patients with psoriasis could activate AIM2 inflammasome in KCs through the p-38 MAPK signaling pathway, inducing IL-1β production. Interestingly, it has already been reported that NET contains mtDNA (32, 33) and that dsDNA stimulation of cultured KCs triggers AIM2 inflammasome–mediated IL-1β release (10). Here, we show that human KC cell line and primary murine epidermal KCs stimulated by dsDNA or Poly(dA:dT) produce the neutrophil-attracting chemokine CXCL1 as well as the inflammasome-dependent IL-18 cytokine. Together, these studies and our findings suggest that dsDNA may activate AIM2 in KCs, promoting the development of psoriasis. However, future studies are required to underline the exact mechanism involved and the potential role of AIM2 in other cell subsets, including immune cells by conditional deficient mice. Moreover, additional innate receptors, including other DNA sensors, also trigger and maintain psoriatic skin inflammation. For example, the LL37 peptide forms a complex with nucleic acids that can activate TLR9 in KCs or plasmacytoid DCs to further promote the production of proinflammatory mediators during psoriasis (34, 35). This could explain why AIM2 deficiency fails to completely mitigate psoriatic skin inflammation.

Finally, we investigated whether AIM2 contributes to psoriasiform skin inflammation through canonical inflammasome activation. After AIM2 oligomerization along DNA strains, AIM2 recruits ASC as an adapter protein, which in turn interacts with caspase-1 through a CARD-CARD interaction, resulting in pro–IL-1β cleavage by caspase-1 (6). We found that genetic ablations of PYCARD or caspase-1 prevented the development of IMQ-induced psoriasis, highlighting a role in the inflammasome. We also directly stimulated AIM2 by intradermal injection of poly(dA:dT) and found that the psoriasiform skin inflammation was reduced in both *Aim2*^–/–^ and *Pycard*^–/–^ mice. Thus, our study demonstrates that AIM2 is required to induce psoriasiform inflammation through an inflammasome-dependent mechanism. In line with our data, another study shows that EFLA 945, a constituent of red grape vine leaf extracts known to prevent DNA entry, attenuates IMQ-induced psoriasis–related proinflammatory responses in topical psoriatic skin, including caspase-1 activation, IL-1β maturation, and IL-17 production, and decreases the severity of psoriasis. Thus, EFLA 945 restricts AIM2 inflammasome activation by preventing DNA entry (36).

In summary, we report that AIM2 is an important immune sensor promoting psoriasiform skin inflammation, amplifying immune cell migration and KC activation/proliferation, and that IL-17A induces its expression in KCs. Furthermore, we demonstrate that AIM2 is involved in psoriasiform inflammation through its canonical activation. Therefore, our findings provide insights regarding the development of psoriasis and strengthen the potential of formulating drugs targeting AIM2 as a new therapeutic strategy for clinical psoriasis management.

## Methods

### Sex as a biological variant.

Sex was not considered as a biological variable. Both male and female mice and patients were included in this study.

### Mice.

We used male and female 8- to 12-week-old C57BL/6 mice, including WT (The Jackson Laboratory, stock no. 000664), *Aim2^–/–^* (37), *Pycard ^–/–^* (38), *Casp1^–/–^* (39), *Il1r^–/–^* (40), and *Il17ra^–/–^* (41). All mice were maintained in specific-pathogen–free conditions at the Ribeirao Preto Medical School under controlled temperature (22°C–25°C), 12-hour light-dark cycle and were provided with water and food ad libitum.

### Patients.

Blood samples were collected from 16 healthy donors and 13 patients with vulgar psoriasis with active psoriasis lesions; criteria for moderated-severe psoriasis involved a body surface area (BSA) of more than 10% and/or Psoriasis Area and Severity Index (PASI) score of more than 10. Human skin samples were obtained from 12 patients with vulgar psoriasis, with a 2 mm punch biopsy, from lesioned or nonlesioned regions. All the samples were used in frozen sections by immunofluorescence.

### Psoriasiform mouse model.

IMQ (5%, Farmoquimica, AR030616) was topically applied daily on the back (60 mg) or ear skin (20 mg) in 8- to 10-week-old mice for the duration indicated in the figure legends (6 days). The mice were previously shaved in the back skin using a shaver and depilatory Veet cream (Silk & Fresh Technology) to remove the residual fur. After 2 days, the IMQ cream was applied to the ear or back skin. Poly(dA:dT) (InvivoGen, tlrl-patn-1, 20 μg) or PBS was injected intradermally daily for 4 consecutive days. The injections were performed using an insulin syringe in a 15 μL/ear volume. The severity of psoriasis was assessed by skin thickness measurement and histological analyses.

### In silico analysis.

We used the GEOquery package (42) to download author-normalized expression data and sample metadata from the following studies: GSE13355 (16), GSE14905 (14), GSE30999 (17), GSE117468 (25), GSE6710 (18), GSE53552 (15). In the studies, the batch effects were detected using scan dates as batch surrogates and then removed using the sva package. Normalization was performed using the Robust Multi Array algorithm from the affy package (42). The limma package (43) was used to identify the differentially expressed genes between patients with healthy, nonlesioned, and lesioned psoriatic skin. The ambiguity between probes that matched the same gene symbol was removed by selecting probes with the highest expression mean between samples. Quality control of microarray samples was performed using the ‘mdp’ package. After normalization, the *AIM2* expression in these different databases was quantified and represented in GraphPad Prism 9 (GraphPad Software).

### Flow cytometry.

The skin was cut into small pieces and digested in RPMI, 5% FCS supplemented with collagenase from *Clostridium histolyticum* (1 mg/mL) and deoxyribonuclease (DNase) I (100 μg/mL; both Sigma-Aldrich) at 37°C for 1.5 hours. After digestion, the skin tissue was disrupted with a syringe with an 18-gauge needle, filtered through a 100 μm cell strainer, and washed with PBS. For surface labeling, the cell suspensions were incubated with antibodies in PBS at 4°C for 30 minutes. The samples were acquired on FACS Verse (BD Biosciences), and the data were analyzed using FlowJo software (Tree Star Inc.).

### Circulation DNA extraction and mtDNA relative quantification by quantitative PCR (qPCR).

For the relative quantification of mtDNA, it was initially performed the extraction of total circulating DNA from the plasma samples of patients using the kit DNA isolation Kit (TIANgen Biotech) following the manufacturer’s instructions. Later, the samples were quantified and evaluated for their quality in NanoDrop One/Onec (Thermo Fisher Scientific). The qPCR method used was adapted from Meddeb et al. (44), and it is based on primers targeting a WT sequence of the KRAS nuclear gene and the mitochondrial Cytochrome oxidase III gene, MT-CO3. Quantification of these amplicons gives an estimation of the nuclear DNA (nDNA) and mtDNA concentrations, respectively. For nDNA, we amplified the KRAS gene with the following primers: forward, 5′-CCTTGGGTTTCAAGTTATATG-3′; and reverse, 5′-CCCTGACATACTCCCAAGGA-3′. For mtDNA, we amplified the cytochrome oxidase subunit 3 mitochondrial genes with the following primers: forward, 5′-GACCCACCAATCACATGC-3′; and reverse, 5′-TGAGAGGGCCCCTGTTAG-3′. The qPCR reaction was performed using 5 ng of total DNA, 20 μM primers, and 1× syber Master Mix (PCRBiosistems). All tests are performed in triplicate in a 10 μL reaction volume on a QuantStudio 5 Real-Time PCR System (Applied Biosystems). The amplification protocol was 95°C for 10 minutes, followed by 40 cycles of 95°C for 15 seconds and 60°C for 1 minute. The relative quantities of transcripts were calculated, and data were represented by fold change between mtDNA and nDNA, respectively.

### RNA extraction and qPCR.

Total RNA from the whole skin was isolated using the RNeasy Kit according to the manufacturer’s instructions (Qiagen). First-strand cDNA was synthesized from 500 μg of mRNA using the high-capacity cDNA Reverse Transcription Kit (4368814, Thermo Fisher Scientific). qPCR was performed using Power Syber Green Master Mix (Thermo Fisher Scientific), primers as described in Supplemental Table 1, and the Viia7 Real-Time PCR system. The data were normalized to *Gapdh* values, and the fold change was analyzed using the DDCt method.

### H&E staining and immunostaining.

The ear and back skins were fixed in 4% paraformaldehyde (PFA) at 4°C for 24 hours and then embedded in paraffin for H&E staining. The skin thickness was measured histologically in a blinded manner. Data represent the mean of 3 measurements on randomly selected areas. Image acquisition was performed on a Leica MMC microscope and analyzed in ImageJ software (NIH). For immunofluorescence staining, human and mouse skin were collected previously in PBS before being fixed in 4% PFA (Sigma-Aldrich) 4°C for 24 hours, washed in PBS, and cryoprotected in 30% sucrose solution (Synth) for 48 hours. Tissues were then embedded in the OCT compound (Tissue-Tek; Sakura Finetek) and snap-frozen on dry ice. Skin sections were cryostat-cut (Leica) into 15 μm–thick transverse sections mounted on glass slides. The samples were then incubated with the working solution (PBS [Corning, 21-031-CV], 1% BSA [Sigma-Aldrich, A7906], 0.05% Tween [Sigma-Aldrich, P2287], 22.52 mg/mL glycine [Sigma-Aldrich, G8898]) at 4°C for 30 minutes. Subsequently, the sections were incubated with primary anti-AIM2 antibody (Abcam, ab93015) diluted in the working solution at 4°C overnight. The sections were washed at least 5 times with PBS-0.05% Tween for 5 minutes before incubating with relevant secondary antibody and DAPI (Abcam, ab285390) diluted in the working solution at 4°C for 30 minutes. The sections were washed at least 5 times with PBS-0.05% Tween, and the mounting medium was added. Image acquisition was performed on an LSM 780 microscope (Carl Zeiss). Images acquired were analyzed using Fiji by ImageJ.

### ELISA.

Cytokine or protein levels in cell supernatants and homogenized tissue samples were measured by ELISA according to the manufacturer’s protocols using the DuoSet ELISA kit (IL-1 β, DY401; IL-17, DY421; IL-23, DY1887; IL-6, DY406; IL-18, DY318-05; IL-8/CXCL-8, DY208; CXCL1/KC, DY208; R&D Systems).

### KC culture in vitro.

Human KCs cell line (HaCaT) was expanded and maintained in DMEM (Thermo Fisher Scientific) culture medium supplemented with 10% FBS, glucose (Thermo Fisher Scientific, 12657-029, 4.5 g/L), penicillin (Sigma-Aldrich , P4333, 100 U/mL), L-glutamine (Thermo Fisher Scientific, 35050061, 2 mM), and gentamycin (Sigma-Aldrich , G1272, 0.2 mg/mL) at 37°C and 5% CO_2_. HaCaT cells were stimulated with IL-17A (100 ng/mL, R&D systems) in a 24-well plate for 24 hours. Subsequently, cells were transfected with dsDNA (500 ng/mL) from the HaCaT itself using Lipofectamine 3000 (Thermo Fisher Scientific) or poly(dA:dT) (InvivoGen, tlrl-patn-1, 500 ng/mL) for 12–16 hours. Twenty-four hours after removing the transfection mix, the cell supernatant was collected for ELISA.

### Culture of primary KCs from neonatal mice.

The culture of primary KCs was done using skin from a pool of 6 neonatal mice (1–3 days after birth). After 4 days in culture, with the renewal of the supplemented medium every day, the cells were primed with IL-17 (100 ng/mL) for 24 hours. Subsequently, cells were transfected with dsDNA (500 ng/mL) from the primary KC itself using Lipofectamine 3000 (Thermo Fisher Scientific) or poly(dA:dT) (InvivoGen, tlrl-patn-1, 500 ng/mL) for 12–16 hours. Twenty-four hours after removing the transfection mix, the cell supernatant was collected for ELISA.

### Statistics.

Statistical analyses were performed using GraphPad Prism 9 (GraphPad Software) and the R studio program. Comparisons for 2 groups were calculated using unpaired 2-tailed Student’s *t* tests and multiple comparisons by 1-way ANOVA or 2-way ANOVA with Bonferroni’s post hoc tests. Correlations were analyzed with 2-tailed nonparametric Spearman rank correlation tests. *P* values below 0.05 were considered significant. *n* represents the number of biological replicates for each experiment that was repeated at least twice at different times. Statistical details for each experiment can be found in the corresponding figure legends.

### Study approval.

Samples collected from patients were previously approved by the Ethics Committee of Ribeirao Preto Clinical Hospital (no. 65644117.1.0000.5440). Informed consent was obtained for all procedures. The animal experiments were carried out in accordance with the guidelines of the Animal Welfare Committee of the Ribeirão Preto Medical School, University of São Paulo (protocol no. 077/2019).

### Data availability.

Public datasets related to this article can be found in the GEO database and are accessible using the accession nos. GSE13355, GSE14905, GSE30999, GSE53552, GSE6710, and GSE117468. The raw data used to make the graphs found in the manuscript’s figures are available in the accompanying Supporting Data Values file.

## Author contributions

TVM and JCAF designed the study. TVM, BMSDM, JETK, CEAS, IMP, GAP, MHR, and GVLDS acquired data. TVM, BMSDM, and GVLDS analyzed data. TVM, FQC, TMC, BR, and JCAF provided funding acquisition. DSZ, CDSS, FQC, TMC, and JCAF provided resources. JCAF contributed project administration. JCAF contributed supervision. Writing of the original draft and preparation were contributed by TVM. Review and editing of the manuscript were contributed by TVM, BR, NR, and JCAF.

## Supplementary Material

Supplemental data

Unedited blot and gel images

Supporting data values

## Version 1 10/01/2024

In-Press Preview

## Version 2 11/08/2024

Electronic publication
