# Supplementary material for: The DNA sensor AIM2 mediates psoriasiform inflammation by inducing type 3 immunity
Source: JCI Insight. 2024 Oct 1;9(21):e171894. doi: 10.1172/jci.insight.171894 (PMC11601563; doi:10.1172/jci.insight.171894)
Supplement: Supplemental data [file jciinsight-9-171894-s180.pdf]

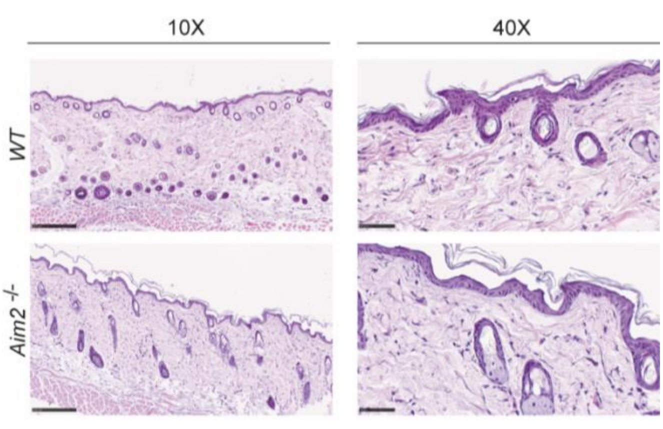

Figure S1: Skin morphology of naïve WT and *Aim2*$^{-/-}$ mice. Representative sections of back skin from naïve WT (C57BL/6) and *Aim2*$^{-/-}$ mice stained with H&E (acquired at 10x and 40x magnification). Scale bars, 100 µm and 50 µm.

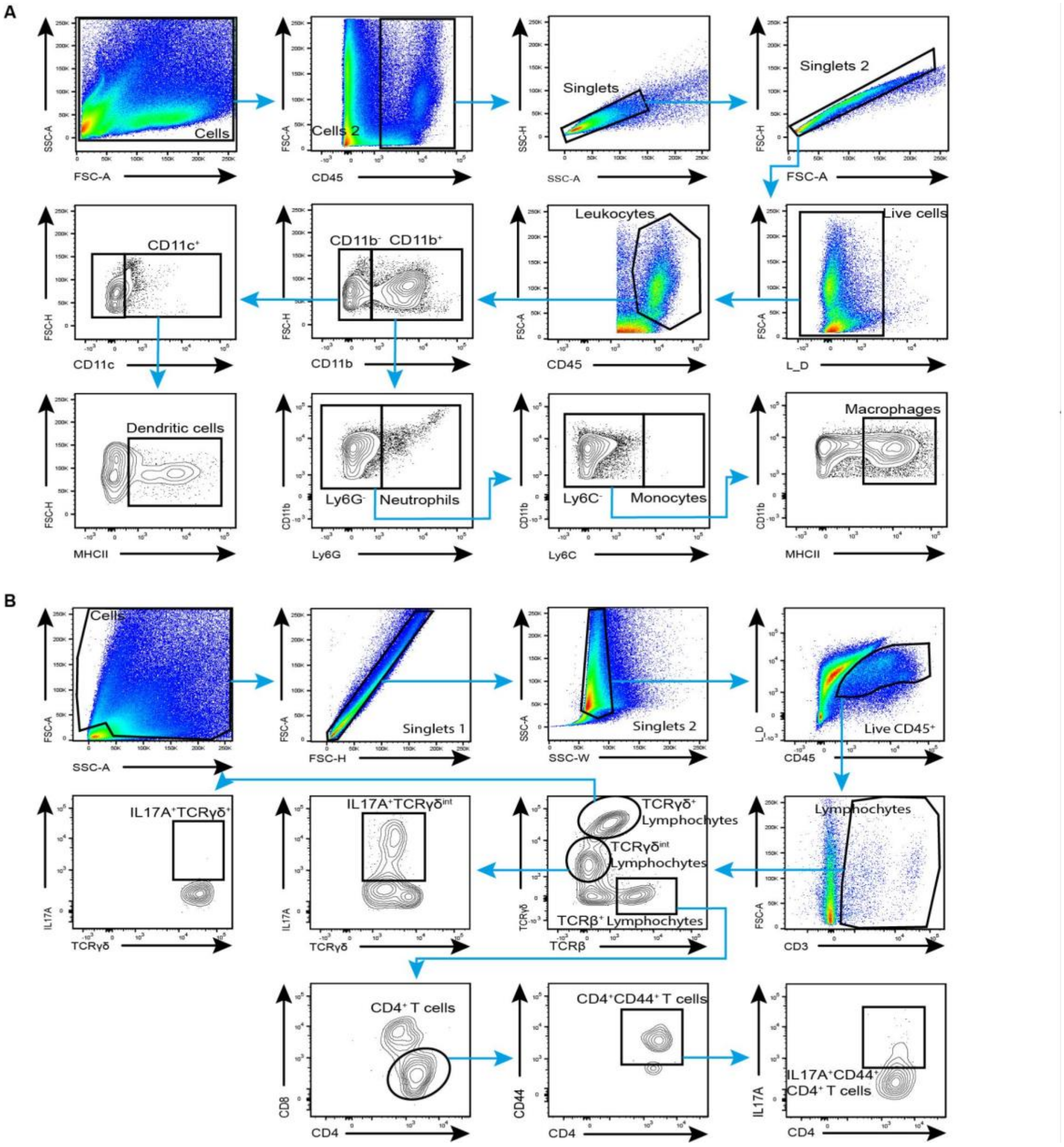

Figure S2: Gating strategy for flow cytometry analyses. (**A**) Representative gating strategy for myeloid cells in the skin. (**B**) Representative gate strategies for lymphoid cells in the skin. All analyzes were performed using Flowjo X software.

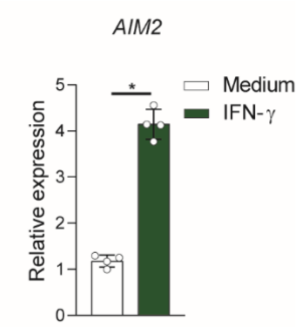

Figure S3: Induction of AIM2 expression in keratinocytes by IFN-γ. Relative mRNA expressions of *AIM2* in HaCaT cells determined by qPCR and normalized to *Gapdh* (n=4 per group). The graph represents the mean ± SEM of data obtained from 2 independent experiments. Statistical analysis was performed by two-tailed unpaired Student's t test, $^*P < 0.05$

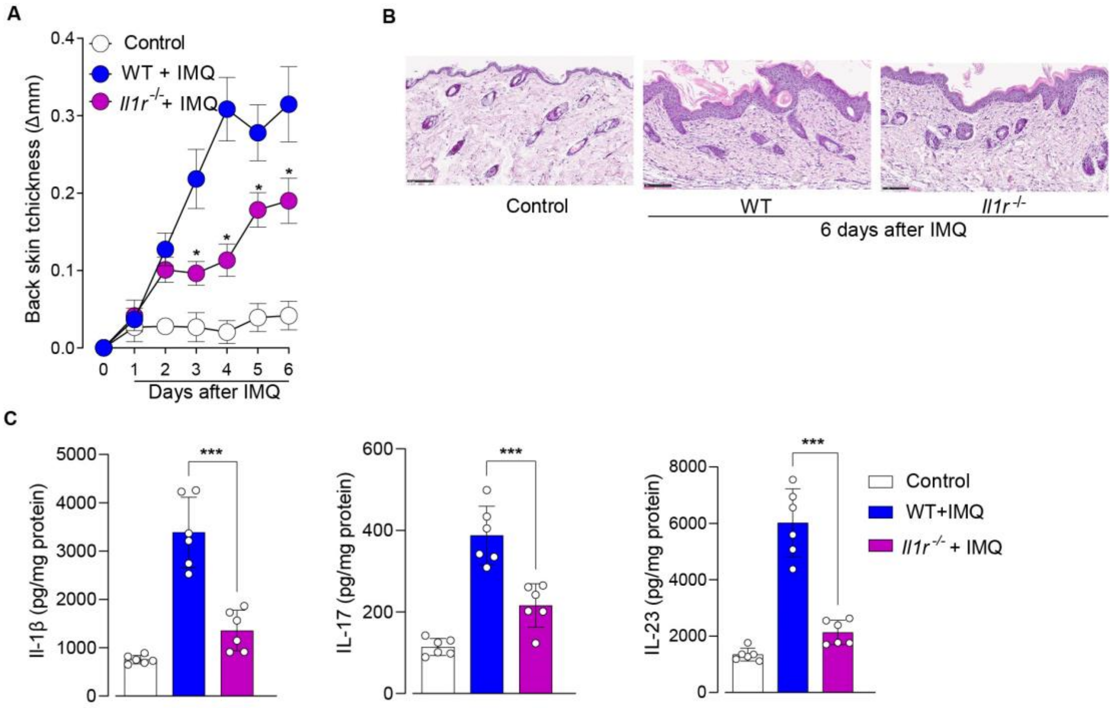

Figure S4: IL-1R is involved in the development of psoriasis-like skin inflammation (**A-C**) WT and *Il1r*$^{-/-}$ mice after 6 days of IMQ treatment. Untreated WT mice were used as controls. (**A**) Time course of changes in the back-skin thickness (delta) measured daily after topical IMQ application by caliper (n=6 per group). (**B**) Representation of hematoxylin and eosin (H&E) staining of back-skin sections of animals. Acquired at 20x magnification. The scale bar indicates 100 µm (n=6 per group). (**C**) IL-1β, IL-23, and IL-17 levels in skin homogenates determined by ELISA (n = 6 per group). Data are representative of 2 independent experiments and are shown as mean ± SEM. Statistical significance was evaluated by two-way (A) or one-way (C) ANOVA followed by Bonferroni's post hoc test, *P < 0.05.

Table S1 - Primers used in this study

KRT14 – Forward	GGCCTGCTGAGATCAAAGACTAC
KRT14 – Reverse	CACTGTGGCTGTGAGAATCTTGTT
Lcn2 – Forward	CCCCATCTCTGCTCACTGTC
Lcn2 – Reverse	TTTTTCTGGACCGCATTG
S100A9 – Forward	GCAGCUGGAACGCAACAUA
S100A9 – Reverse	UAUGUUGCGUUCCAGCUGC
Caspase 1 – Forward	GGCACATTTCCAGGACTGACTG
Caspase 1 – Reverse	GCAAGACGTGTACGAGTGGTTG
AIM2 – Forward	AGGCTGCTACAGAAGTCTGTCC
AIM2– Reverse	TCAGCACCGTGACAACAAGTGG
IL-1 beta – Forward	GAAATGCCACCTTTTGACAGTG
Il-1 beta – Reverse	TGGATGCTCTCATCAGGACAG
