# Supplementary figures and images for: The DNA sensor AIM2 mediates psoriasiform inflammation by inducing type 3 immunity
Source: JCI Insight. 2024 Oct 1;9(21):e171894. doi: 10.1172/jci.insight.171894 (PMC11601563; doi:10.1172/jci.insight.171894)

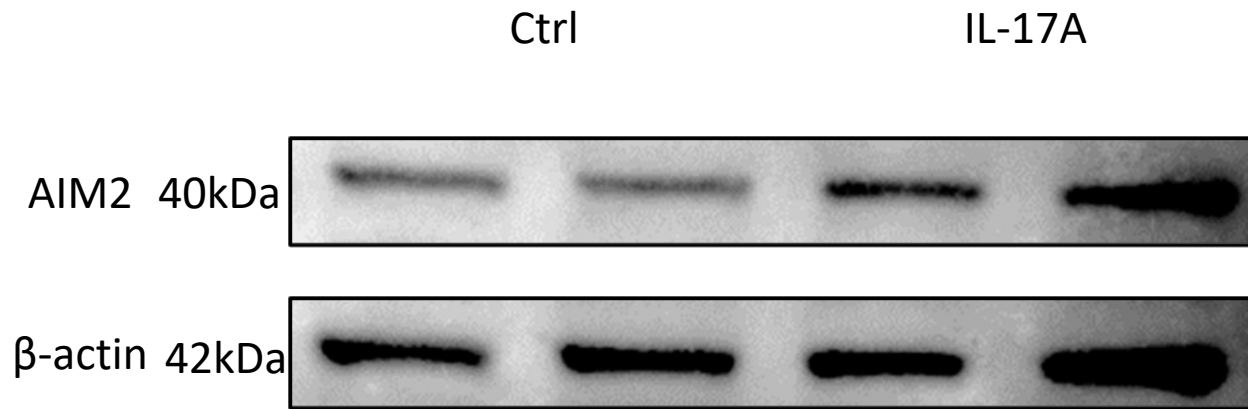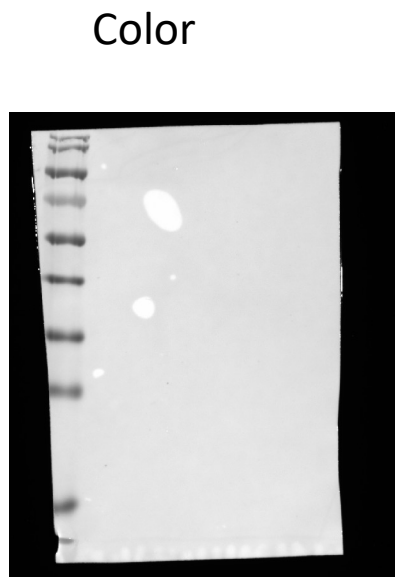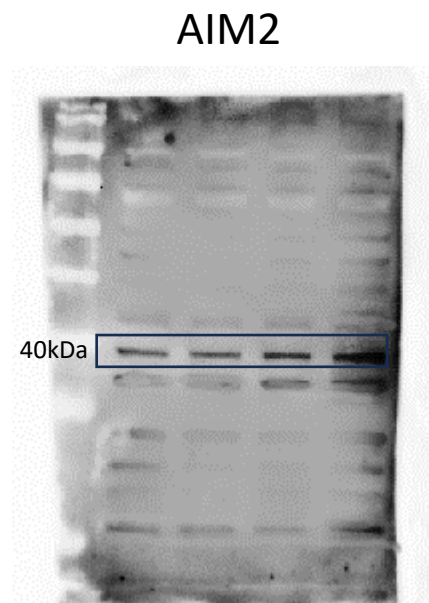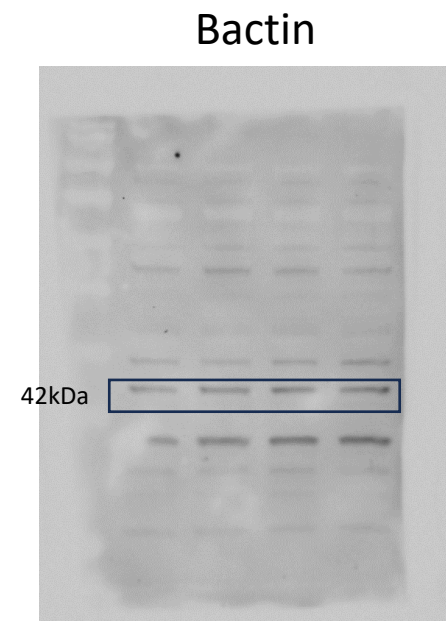

Supplement: Unedited blot and gel images [file jciinsight-9-171894-s181.pdf]
